# Molecular Imaging Using Cardiac PET/CT: Opportunities to Harmonize Diagnosis and Therapy

**DOI:** 10.1007/s11886-021-01526-y

**Published:** 2021-07-01

**Authors:** James T. Thackeray

**Affiliations:** Department of Nuclear Medicine, Hannover Medical School, Neuberg-Str. 1, D-30625 Hannover, Germany

**Keywords:** Molecular imaging, Myocardial infarction, Heart failure, Inflammation, Cardiac fibroblasts

## Abstract

**Purpose of Review:**

Current therapeutic strategies to mitigate heart failure progression after myocardial infarction involve support of endogenous repair through molecular targets. The capacity for repair varies greatly between individuals. In this review, we will assess how cardiac PET/CT enables precise characterization of early pathogenetic processes which govern ventricle remodeling and progression to heart failure.

**Recent Findings:**

Inflammation in the first days after myocardial infarction predicts subsequent functional decline and can influence therapy decisions. The expansion of anti-inflammatory approaches to improve outcomes after myocardial infarction may benefit from noninvasive characterization using imaging. Novel probes also allow visualization of fibroblast transdifferentiation and activation, as a precursor to ventricle remodeling.

**Summary:**

The expanding arsenal of molecular imaging agents in parallel with new treatment options provides opportunity to harmonize diagnostic imaging with precision therapy.

## Introduction

Reperfusion and standard drug therapy have reduced mortality after acute myocardial infarction, but with increased survival more patients are at risk of adverse ventricular remodeling and heart failure development [1, 2]. Standard drug therapies aim to limit damage, generally targeting later manifestations of ventricle remodeling. Paradigm-shifting experiments have illuminated the role of early pathogenetic processes in promoting ventricle remodeling [3, 4], leading to the development of novel molecular-based therapies to promote repair and regeneration of the heart. Initial enthusiasm for cell-based therapy to regenerate damaged myocardium has been dampened due to lack of engraftment and differentiation of transplanted cells [5, 6]. In fact, the observed benefits largely derived from paracrine effects mediating the endogenous healing process [7]. Accordingly, novel therapeutic approaches have evolved to address early manifestations of disease to attenuate or prevent adverse remodeling and promote endogenous tissue repair [8]. The efficacy of natural healing varies between individuals, which ultimately influences the progression of heart failure and the optimal therapeutic approach. Molecular imaging using cardiac positron emission tomography (PET) and computed tomography (CT) affords the capacity to visualize disease progression and assess the efficacy of such therapies [2].

Cardiac PET/CT has established a critical role for superior regional and quantitative evaluation of physiological processes. The predominant clinical application of cardiac PET/CT has been in assessing myocardial blood flow and viability, which provide information on initial damage and response to conventional therapy. This approach generally relies on conducting PET/CT interrogation after therapy has been initiated. Alternatively, direct labeling strategies to track the therapeutic agent at the time of administration can provide added value in drug development. But the greatest potential of PET/CT molecular imaging in cardiac patient management is in pseudo-theragnostics, where the imaging target overlaps with the therapeutic target allowing patient risk stratification, patient selection for precise therapy, and refinement of the dosing and timing of such compounds [2, 9, 10]. While still in its infancy in cardiology, such approaches have the potential to establish cardiac PET/CT as a central contributor to precision medicine.

In this review, we will examine the growing role of cardiac PET/CT to image tissue repair, encompassing the evaluation of physiologic response, direct labeling of therapeutic agents to define distribution and duration of effect, and quantitative imaging of early pathogenetic processes to predict functional decline and guide therapeutic intervention. We will then expound upon the current opportunities in this field of research and define the critical challenges presented by this amalgamation between molecular imaging and precision medicine for effective translation to clinical practice.

## Conventional Imaging to Evaluate Response

Clinical cardiac PET/CT is most commonly used to identify the severity of functional impairment and predict disease progression, with particular focus on defining left ventricle geometry and contractile function as well as quantitative blood flow and flow reserve. Derivation of left ventricle ejection fraction provides a powerful prediction of mortality and progression of heart failure. Quantification of myocardial blood flow and flow reserve also offers added value in identifying risk of heart failure progression [11–13]. Perfusion imaging may be combined with assessment of myocardial viability, using the glucose analogue ^18^F-fluorodeoxyglucose (FDG).

Image guidance in nuclear cardiology has often been relegated to measuring the physiologic response to therapy, via restoration of perfusion or viability [14, 15]. This conventional imaging approach has confirmed the importance of viability for successful reperfusion [16, 17], and the efficacy of novel gene transfer of vascular endothelial growth factor to promote angiogenesis after infarction [18].

However, image-based measurements of perfusion, scar, and function are distant from the novel therapeutic targets aiming to enhance cardiac repair, thereby rendering an imprecise characterization of the molecular biology underlying recovery. As such, while these conventional imaging approaches can define repsonse to initiated therapy, they provide limited opportunity to select patients most likely to respond to molecular-targeted therapy or to directly assess the molecular response to treatment.

## Direct Labeling to Track Therapeutics

A second application of imaging for cardiac repair lies in the capacity to directly monitor the distribution of a therapeutic agent in the body. This concept is exemplified in early stem cell therapy studies, where direct labeling or PET reporter gene approaches allowed short-term and extended visualization of the distribution and limited engraftment of administered therapeutic cells [19, 20]. Despite early promise in preclinical models, the clinical benefit of hematopoietic cell transfer is largely underwhelming [6], with the observed therapeutic benefit deriving almost entirely from modulation of the endogenous inflammatory response [7].

The same reporter genes may be deployed in concert with gene therapy. Combining the SPECT reporter gene sodium iodide symporter (NIS) with adeno-associated virus serotype-9, gene transfer was visualized in the gastrocnemius muscle after unilateral hindlimb ischemia. Uptake of ^99m^Tc-pertechnetate correlated with the adenoviral transfer of NIS to the affected muscle over 28 days after gene therapy [21], suggesting stable gene transfer to myocytes. This study established the feasibility to assess efficacy of gene therapy and the penetrance of the transferred gene. Combination of NIS with a therapeutic gene in the viral vector could allow more relevant measurement of gene transfer and stability, though the stoichiometry for image detection and therapeutic effect likely differ [22]. Nonetheless, similar approaches combining PET reporters with higher spatial resolution could refine such surrogate imaging measures.

The growth “click” chemistry for rapid and efficient labeling in mild conditions has further facilitated the development of new labeling techniques in radiochemistry. New techniques including site-specific bioconjugation, novel chelator construction, and pre-targeting offer new avenues for imaging which have not been extensively explored in cardiac PET/CT [23, 24]. These chemistry approaches may be applied to nanoparticles, antibodies or antibody fragments, or other small molecules, targeting specific components of pathophysiology. One example is the capacity to target micro-RNAs, conjugating oligonucleotide sequences with radiometal chelator for visualization of micro-RNA upregulation in disease. Proof-of-concept studies displayed extended retention in bones and bone marrow of healthy rats [25], but high background signal and poor clearance remain at issue. The potential of micro-RNA-based therapies provides an opportunity for complementary molecular imaging [26]. Furthermore, the challenges of antibody-based imaging, chiefly the long circulation time before clearance from blood contributing to high background signal and poor target-to-noise ratio, may be circumvented in part by truncation of the antibody structure to micro- or nano-bodies, which has shown promise in tumor imaging [27]. Future developments may facilitate more precise targeting of pathogenetic pathways in parallel with the increased utilization of monoclonal antibody therapies in cardiovascular disease [28].

Such chemistry developments and imaging strategies offer the capability to track the distribution of a therapeutic agent during administration and for a short or extended period afterward. These imaging observations can provide valuable insights into drug-target specificity, duration of retention, and off-target effects. But they remain ineffective for identifying patients most likely to benefit from a given treatment.

## Molecular Targets for Imaging and Therapy

**Fig. 1:**
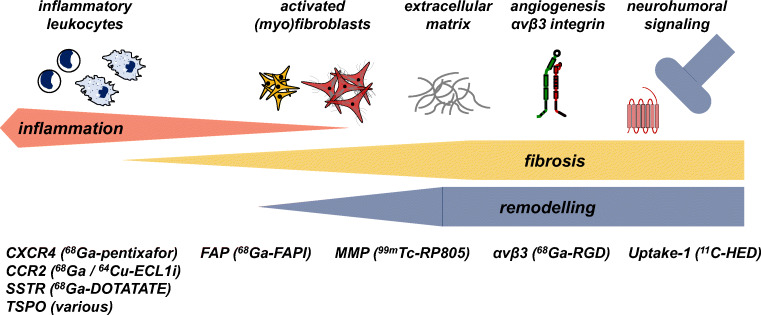
Overview of molecular imaging targets for PET/CT assessment of cardiac repair, covering the spectrum from acute inflammation, replacement and reactive fibrosis, and remodeling including angiogenesis and sympathetic neuronal activation. Early inflammation may be imaged at viable therapeutic targets expressed by infiltrating leukocytes including chemokine receptors (CXCR4, CCR2), somatostatin receptor (SSTR), or mitochondrial translocator protein (TSPO). Transdifferentiation and activation of cardiac fibroblasts can be identified through fibroblast activation protein (FAP). Subsequent reorganization of extracellular matrix can be imaged by targeting matrix metalloproteinases (MMP). Other remodeling processes may be imaged with PET/CT including αvβ3 integrin via binding the RGD peptide sequence or sympathetic neuronal varicosities using norepinephrine analogues such as ^11^C-hydroxyephedrine (HED)

The greatest potential for cardiac PET/CT lies in the capacity to quantitatively image individual variation in pathogenesis at the molecular level. Early and crucial processes including inflammation and myofibroblast activation dictate the severity of ventricle remodeling and likelihood of progressive heart failure (Fig. 1). As such, these processes constitute optimal biomarkers for guiding therapeutic intervention (Table 1), providing unique insight into patients most likely to benefit from matched targeted molecular therapy and expediently identifying therapeutic success.

**Table 1:** Molecular imaging targets for cardiac PET/CT

Pathogenesis	Tracer	Cellular and molecular target
Inflammation	^18^F-FDG	Macrophages
	^18^F-GE180, ^11^C-PK1195, ^18^F-DPA	TSPO in macrophages, central microglia
	^68^Ga-PentixaFor	CXCR4+ leukocytes (neutrophils, macrophages, monocytes, lymphocytes)
	^64^Cu/^68^Ga-ECL1i	CCR2+ monocytes
Fibrosis	^68^Ga-RGD	αvβ3 integrin+ fibroblasts, endothelial cells
	RP805	Matrix metalloproteinases
	^68^Ga-FAPI	Fibroblast activation protein on myofibroblasts
Innervation	^11^C-HED, ^11^C-epinephrine	Uptake-1 on presynaptic sympathetic neurons
Angiogenesis	^68^Ga-RGD	αvβ3 integrin+ neovasculature, endothelial cells
Thrombosis	^18^F-GP1	Glycoprotein IIb/IIIa+ activated platelets

## Imaging Inflammation

Inflammation is typically visualized using ^18^F-FDG, taking advantage of the elevated glucose metabolism of activated inflammatory cells especially macrophages. In vitro studies have established preferential accumulation of ^18^F-FDG by pro-inflammatory macrophage subtypes [29, 30], and gene expression studies have confirmed heightened expression of glucose transporters [31].

While ^18^F-FDG is widely available and boasts a successful application history, cardiomyocytes—especially under duress—avidly metabolize glucose, creating a complex substrate for quantitative imaging. This complication necessitates measures to suppress cardiomyocyte glucose metabolism including extended fasting, high fat loading, and/or administration of heparin to increase circulating non-esterified fatty acids and shift myocardial energy substrate preference toward lipids [32]. In mice, the standard suppression method utilizes ketamine-xylazine anesthesia to inhibit pancreatic insulin secretion in response to rising glucose levels, resulting in a hyperglycemic state [33]. But these suppression methods are not physiological, and not always effective in states of perturbed metabolism such as after acute infarction, which can complicate the interpretation of these images [34, 35]. Accordingly, alternative tracers to better identify inflammatory signals have been widely deployed in preclinical investigations in recent years [29].

### Translocator Protein TSPO

The mitochondrial 18kDa translocator protein (TSPO) is expressed in the outer mitochondrial membrane in various mitochondria-rich cells, including activated microglia in the central nervous system and macrophages in the periphery. This high expression pattern in activated inflammatory cells renders TSPO an attractive imaging target. Numerous compounds have been developed for imaging TSPO with a focus on neuroinflammation, including ^11^C-PK11195, ^18^F-GE180, and ^18^F-DPA-714 [36, 37]. TSPO imaging has identified vascular inflammation in atherosclerosis, with potential added value in identifying high-risk inflamed plaque in direct comparison with ^18^F-FDG [38–40]. In the heart, the high TSPO expression by mitochondria-rich cardiomyocytes hinders widespread application for cardiac injury, though some preclinical studies have shown PET signal enrichment against the cardiomyocyte background signal. For example, increased ^18^F-DPA-714 binding was reported in mouse hearts after transplanted stem cell–derived cardiomyocyte sheets, reflecting the innate and adaptive immune response via macrophages and CD3+ T-cells at the site of transplantation [41]. After coronary artery ligation in mice, elevated TSPO signal relative to perfusion was identified in the infarct territory at 3 and 7 days [42, 43]. Immunostaining for TSPO displayed colocalization to CD68+ macrophages in the infarct and border zone regions. The severity of inflammation at 7 days after myocardial infarction independently predicted functional decline 8 weeks later [43]. These observations were recapitulated in a small group of patients imaged with ^11^C-PK11195 at 4−6 days after myocardial infarction, where increased tracer binding was localized to the perfusion defect [43]. Nonetheless, the capacity to simultaneously monitor peripheral and central nervous system inflammation with one compound yields high potential, particularly in the context of multi-organ analyses.

### Chemokine Receptor CXCR4

The expansion of the inflammation-targeted radiotracer armamentarium can improve quantitative definition of the inflammatory signal, providing added value in clinical care. Chemokines secreted at the site of injury attract inflammatory cells through interaction with specific receptors expressed on the leukocyte surface. Different types of chemokine receptors are found on various leukocyte subtypes: e.g., chemokine CC motif receptor 2 (CCR2) is expressed primarily on infiltrative monocytes and derivative macrophages, and chemokine CXC motif receptor 4 (CXCR4) is expressed on a wider range of cells including granulocytes, multiple macrophage varieties, and lymphocytes.

**Fig. 2:**
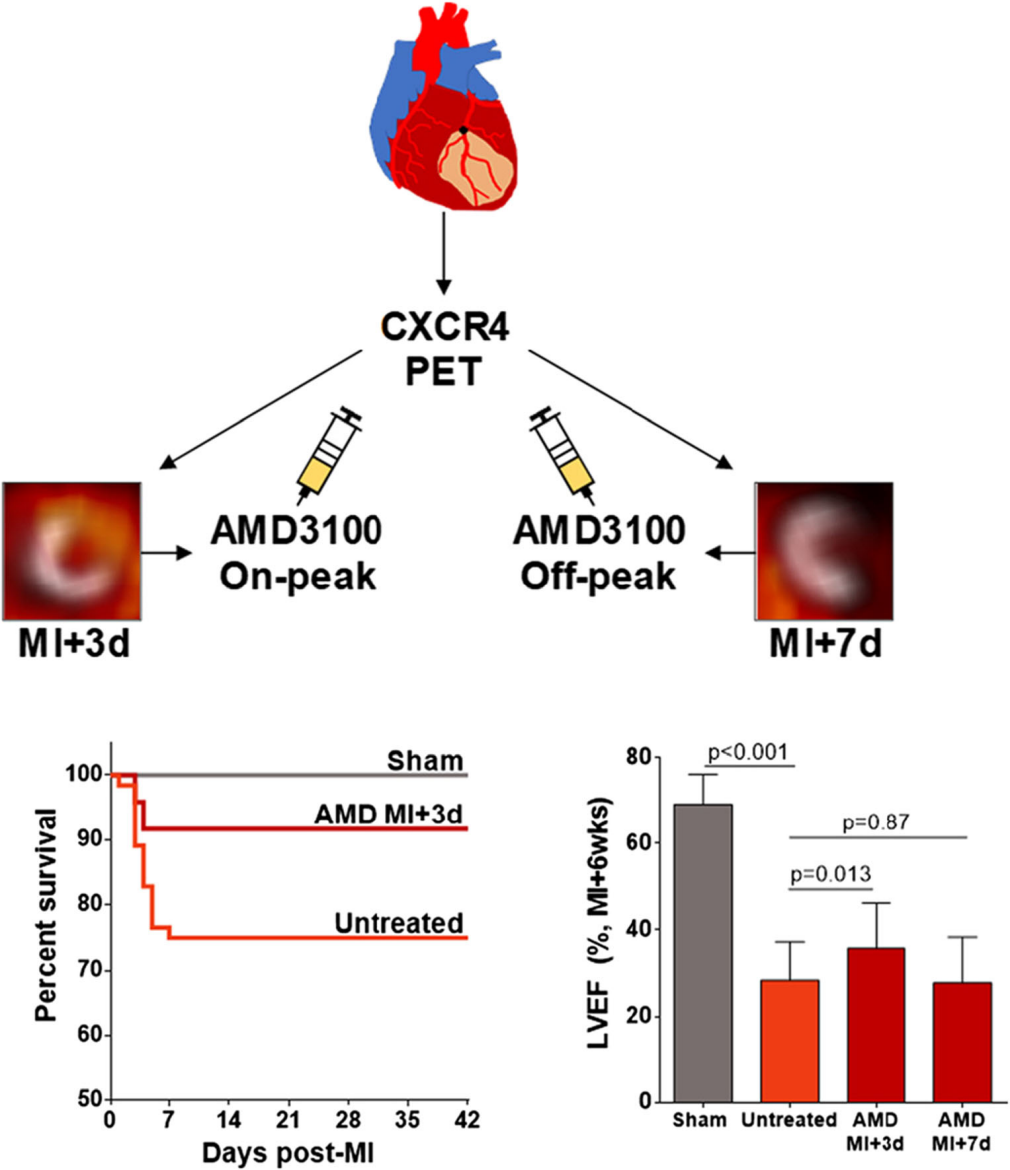
Concept and response of PET/CT imaging–guided anti-inflammatory therapy at the chemokine receptor CXCR4. Serial imaging after myocardial infarction (MI) in mice identified early increase in the CXCR PET signal (colorscale) in the infarct territory defined by FDG (grayscale) at 3 days after injury that predicted contractile function 6 weeks later. Single-time treatment with a selective CXCR4 inhibitor (AMD3100) at the time of elevated signal (3 days) resulted in lower incidence of acute ventricle rupture (lower left panel) and improved contractile function at 6 weeks (lower right panel) compared to untreated MI or treatment at a time without elevated CXCR4 PET signal (7 days) (Reproduced from Hess et al., with permission from Oxford University Press [44••])

Imaging using the CXCR4 ligand ^68^Ga-pentixafor in mice after permanent left coronary artery ligation revealed rapid and transient increase in total leukocyte content in the infarct territory. The intensity of the PET imaging signal at 3 days after injury predicted the severity of ventricle remodeling and contractile dysfunction 6 weeks later, independent of the infarct size. Moreover, mice with prolonged upregulation of CXCR4 over 3 days after ischemic injury had a higher incidence of acute ventricle rupture [44••]. Patients after acute infarction exhibit variable CXCR4 signal [45, 46], but the imaging signal obtained 4−6 days after reperfused myocardial infarction also correlates with contractile function 6−9 months later [44••]. That is, patients with higher CXCR4 content in the left ventricle have worse outcomes than patients with lower expression patterns. Critically, single-time administration of a selective inhibitor of CXCR4 at the time of elevated and predictive PET signal prevented ventricle rupture and improved chronic contractile function among surviving mice, whereas treatment at a time distant from peak PET signal bore no benefit [44••]. This study illustrates the capacity to couple imaging with therapeutic targets to refine the treatment strategy for optimal benefit (Fig. 2).

### Chemokine Receptor CCR2

Crucial lineage tracing experiments have highlighted the unique contributions of tissue-resident macrophages and those derived from infiltrating monocytes [47]. Expression of the chemokine receptor CCR2 identifies early infiltrating bone marrow–derived monocytes which differentiate to inflammatory CCR2+ macrophages at the site of infarction, where they aggravate local tissue inflammation contributing to heart failure [47]. As such, CCR2+ macrophages represent a potential therapeutic target to modulate acute inflammation in a specific manner. To visualize CCR2 in vivo, a peptide-based radioligand ^64^Cu-DOTA-extracellular loop 1 inverso (ECL1i) has been developed. Transgenic mice expressing diphtheria toxin receptor under a troponin T2 promoter allowed cardiomyocyte ablation via diphtheria toxin administration, generating local myocyte damage. ^64^Cu-DOTA-ECL1i uptake selectively accumulated in the damaged heart, which directly correlated with pro-inflammatory cytokine interleukin-1β and interleukin-6 levels [48•]. Likewise, after myocardial infarction in mice, the CCR2 PET signal enhanced in the infarct region, reaching maximal signal at 4 days after coronary artery occlusion and returning to sham levels by 2 weeks. Tracer uptake directly correlated with CCR2+ cells isolated by fluorescence-associated cell sorting [48•]. As for CXCR4, the signal intensity described at 4 days was predictive of subsequent contractile function at 4 weeks after infarction. The translational potential of this approach was further tested by autoradiography in human heart sections, where tracer uptake was highest in the acute infarct tissue, which was effectively blocked by unlabeled compound [48•]. Subsequent labeling with gallium-68 demonstrated similar tracer kinetics and distribution, supporting wider application without dependence on cyclotron production [49]. Initial clinical investigations in pulmonary fibrosis demonstrate favorable dosimetry and toxicity profiles [50], potentially facilitating translation to clinical application. The development of novel therapeutics against CCR2 which may mediate the acute inflammatory response may therefore be effectively monitored or guided through molecular imaging approaches.

## Imaging Fibrosis

Fibrosis is the excessive accumulation of extracellular matrix typically found in the remodeling left ventricle, resulting from activation of cardiac fibroblasts. Acute ischemic injury stimulates transdifferentiation of quiescent fibroblasts. These active (myo)fibroblasts migrate to the area of damage to facilitate scar formation via replacement fibrosis [51, 52]. In the failing heart, activation of fibroblasts secondary to a myriad of factors including cardiac stress, pressure- or volume-overload, or cardiomyopathy, leads to reorganization of extracellular matrix in the interstitial space between cardiomyocytes, or reactive fibrosis. The resulting increased ventricle stiffness stimulates further ventricle remodeling and progression of heart failure [52, 53].

PET/CT imaging has visualized extracellular matrix reorganization by targeting the effector enzymes matrix metalloproteinases. Based on profiling studies, matrix metalloproteinases were found to be upregulated in acute coronary syndrome and after myocardial infarction [54, 55], driving interest in developing broad-spectrum inhibitors to mitigate ventricle remodeling [56, 57]. Compounds to image matrix metalloproteinases include several SPECT agents such as ^111^In-RP782 or ^99m^Tc-RP805, which accumulate in remodeling vasculature and myocardium [58–61]. The intensity of the imaging signal predicts adverse outcomes and responds to targeted matrix metalloproteinase inhibition [61]. However, matrix remodeling is a relatively late pathogenetic process in ischemic heart failure progression, which may restrict the benefit for many patients.

Selective identification of early fibroblast activation has posed challenges in preclinical and clinical studies. Early characterization of fibrosis by PET/CT was pursued by targeting the αvβ3 integrin, which is expressed by activated fibroblasts. Initial preclinical and clinical studies indicated that arginine-glycine-aspartic acid (RGD) peptide imaging identified myofibroblasts in the infarct region in rodents and humans [62–64]. Unfortunately, αvβ3 integrin is also strongly expressed by macrophages and vascular endothelial cells [65–67], complicating interpretation of the imaging signal.

Accordingly, more specific imaging agents targeting fibroblasts have been desirable. A promising target for molecular imaging is the fibroblast activation protein, which exhibits low expression by quiescent cardiac fibroblasts, but is rapidly upregulated in response to injury stimuli, and during fibroblast transdifferentiation [68]. Notably, FAP was also identified as an oncologic imaging target for tumor fibrosis, and a series of imaging agents have been developed for the imaging of cancer-associated fibroblasts [69].

**Fig. 3:**
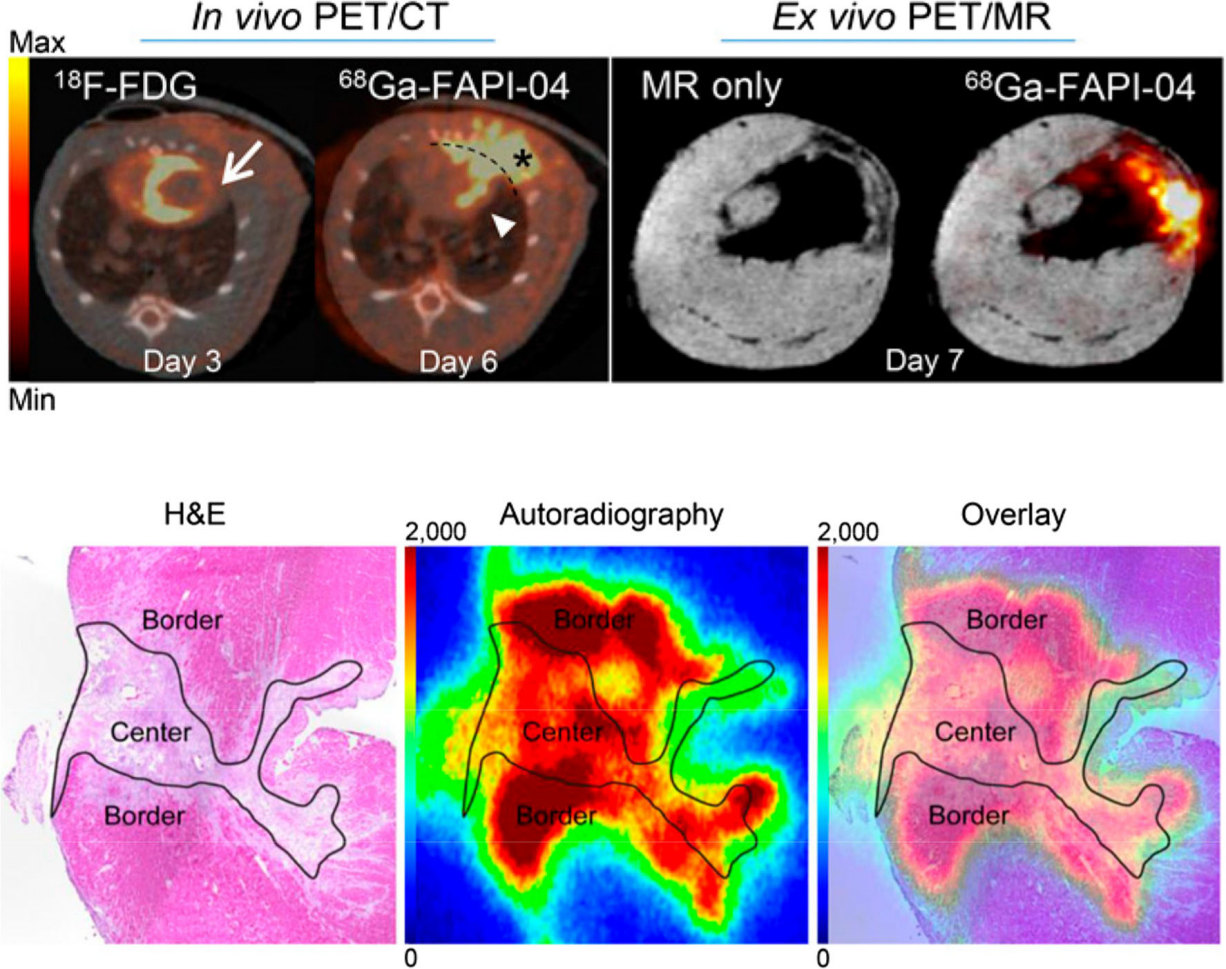
Imaging of activated myofibroblasts after myocardial infarction in rats using the fibroblast activation protein (FAP) targeted ^68^Ga-FAPI-04. At 6 days after coronary artery occlusion, FAPI PET signal is increased in the infarct and border zone territory defined by reduced 18F-FDG uptake (upper panel, left) and confirmed ex vivo by PET/MR (upper panel, right). High-resolution autoradiography and adjacent hematoxylin and eosin (H&E) histology localized the strongest FAPI signal to the border zone surrounding the center of the infarct (lower panel) (Reproduced from Varasteh et al., with permission from JNM [70•])

Serial imaging in rats after ligation of the left anterior descending coronary artery displayed a gradual increase in ^68^Ga-FAPI-04 binding in the infarct and border zone regions, reaching a maximal signal at 6 days after surgery. Ex vivo PET/MR imaging and autoradiography localized the accumulation of the FAP-targeted tracer to the damaged region (Fig. 3). The site of the surgical wound also exhibited a significant FAPI signal from the skin and surrounding area, raising questions as to the specificity of the radiotracer for cardiac myofibroblasts. Fluorescence co-immunostaining delineated colocalization of FAP with prolyl-4-hydroxylase and vimentin, markers of activated fibroblasts, reflecting myofibroblasts, and limited colocalization to mature fibroblasts denoted α-smooth muscle actin [70•]. These promising preclinical results have spurred interest for clinical translation of FAP imaging.

Retrospective analysis of myocardial ^68^Ga-FAPI uptake in a mixed oncology patient population revealed an association between higher signal intensity and cardiovascular risk factors. The presence of more cardiovascular risk factors, diagnosed coronary artery disease, and arterial hypertension were all associated with higher FAPI signal from the left ventricle, particularly in the interventricular septum. Likewise, rising body mass index or diabetes mellitus corresponded to higher signal intensity in the myocardium. The authors verified the initial findings in a confirmatory cohort, illustrating the potential value of FAPI imaging to identify early-stage cardiovascular disease [71]. A separate retrospective study identified localized myocardial uptake in 6/32 patients after FAPI imaging for cancer staging. This localized uptake in non-infarcted remote myocardium significantly correlated with coronary artery disease, age, and ejection fraction [72]. Naturally, the retrospective design limits the conclusions that can be drawn, and prospective cardiovascular cohorts will be necessary to determine the added value of FAPI imaging for prediction of functional outcome. Case reports in anthracycline-induced cardiotoxicity and cardiac angiosarcoma provide some indication of the capacity to image myocardial fibroblast activation [73, 74]. But dedicated prospective clinical studies will be necessary to glean the added value to this imaging process.

## Other Molecular Imaging Targets

Beyond inflammation and fibrosis, other pathophysiological processes have been targeted for image-based assessment of cardiac repair. Central to these is interrogation of the sympathetic nervous system, the primary extrinsic control of heart rate and contractility.

The cardiac sympathetic nervous system remains of interest for molecular imaging, due to the direct relationship between sympathetic activity, cardiomyocyte β-adrenoceptor expression, and ventricle remodeling. In the failing heart, increased sympathetic drive leads to increased norepinephrine stimulation and downregulation of adrenoceptors, the pathophysiology underlying common use of β-blocker therapy to normalize contractility. Imaging of sympathetic nervous activity depends largely on radiolabeled analogues of norepinephrine such as for which neuronal retention correlates directly with sympathetic neuronal density, and inversely with synaptic norepinephrine content [75]. In heart failure patients, the extent of denervation denoted by ^11^C-meta-hydroxyephedrine retention predicts sudden cardiac arrest, but more sophisticated quantification provided limited additional predictive value [76, 77]. However, sympathetic neuronal imaging in cardiac PET/CT has been largely stagnated, owing in part to reliance on carbon-11 chemistry which necessitates an on-site cyclotron. The development of a fluorine-18-labeled norepinephrine analogue with favorable kinetics, ^18^F-LMI1195, may facilitate wider application [78]. Preliminary quantitative analysis appears to reflect the established parameters of ^11^C-meta-hydroxyephedrine, such that the existing experience may be applied to the new compound. The second major limitation for sympathetic neuronal imaging, however, cannot be addressed with an alternative label. Unlike inflammation and fibroblast activation, the molecular imaging target imaging target is distinct from the therapeutic target (i.e., presynaptic uptake-1 versus postsynaptic adrenoceptors). While experimental evidence shows parallel regulation of the pre- and postsynaptic targets [79, 80], the added value in monitoring or guiding therapy is necessarily limited due to the effective prescription of beta blocker therapy and lack of direct connection between the imaging target and device therapy.

Recent studies have also highlighted alternative molecular targets that exhibit cardioprotective effects and are emerging therapeutic targets. Glucagon-like peptide 1 (GLP-1) signaling is cardioprotective in diabetic patients [81] and counteracts adverse remodeling after myocardial infarction [82]. Recent studies have shown promise of imaging GLP-1 receptor to predict functional outcome. Immunostaining demonstrated increased GLP-1 receptor binding in the infarct region early after coronary artery ligation rats, corresponding to CD68+ macrophage content. Modest increase of in vivo imaging signal using ^68^Ga-NODAGA-exendin was identified at 3 days, receding dramatically by 1 week, and back to baseline levels at 12 weeks after injury. Kinetic modeling suggested irreversible binding at the early timepoint which was absent in later stages. Likewise, autoradiography signal showed enrichment of ^68^Ga-NODAGA-exendin binding in the infarct region up to 12 weeks. The incongruence of immunostaining and imaging signal questions the selectivity of exendin for GLP-1R, but binding kinetics described a significant correlation to CD68+ area in the infarct territory, and α-smooth muscle actin content in the remote myocardium [83]. It remains unclear whether exendin-directed therapy may benefit the heart, but an indirect indicator of inflammation may provide additional insights into disease progression.

Several other compounds have been developed with an eye to imaging and therapy including those targeting thrombosis [84, 85], angiogenesis [86, 87], and angiotensin signaling [88]. But the best-characterized imaging targets most likely to impact patient care remain inflammation and fibroblast activation.

## Challenges and Opportunities

As more molecular imaging options become available, it will be essential to effectively identify high potential candidate tracers and incorporate imaging to optimize and refine reparative therapy. The characterization of such tracers should have distinct characteristics to maximize potential clinical impact (Table 2). First, the molecular targets of imaging agents should be closely linked to a viable therapeutic target, harmonizing the imaging readout with the direct effects of the drug or treatment. This approach allows for more precise identification of patients most likely to benefit from targeted treatment, monitor the early response to therapy, and guide long-term intervention. Second, preclinical studies should demonstrate the capacity to define the risk of disease progression. That is, studies should take advantage of quantitative nature of PET to relate tracer uptake with prognostic outcome. This approach requires long-term and serial imaging studies in animals and subsequently in patients to define the added value to standard patient care. Third, the optimal tracer must be sufficiently sensitive to indicate therapeutic response early after the intervention, allowing refinement of dosing or discontinuation of ineffective therapy. As such, early experiments must demonstrate the ability to differentiate subtle changes in the biomarker expression, which in turn enhances the prognostic information provided by PET/CT. Taken together, these approaches enable PET/CT imaging to evolve from a bystander readout of functional response to an active participant in patient management.

**Table 2:** Challenges and opportunities to harmonize diagnosis and therapy

Challenge	Opportunity and future direction
Harmonize targets	- Development of radiotracers to targets with corresponding drugs - Definition of individual patient expression to guide therapy
Define prognosis	- Preclinical and clinical definition of prognostic impact of imaging - Follow-up studies to define the clinical relevance of the biomarker
Assess response	- Sensitivity of imaging marker to therapy to define efficacy - Refine treatment strategies based on imaging expression profile

## Conclusion

Growth and further development of novel molecular imaging agents stand poised to revolutionize PET/CT imaging in the heart, moving beyond conventional assessment of latter-stage pathology toward the underlying molecular mechanisms. The closer link between imaging and therapeutic targets provides the unique opportunity for precision interrogation of pathogenesis and response to therapy. Accordingly, cardiac PET/CT can harmonize its established role in sensitive diagnosis with the future prospect of exquisitely guided molecular therapy.
